# Core needle biopsies alter the amounts of CCR5, Siglec-15, and PD-L1 positivities in breast carcinoma

**DOI:** 10.1007/s00428-023-03563-0

**Published:** 2023-05-24

**Authors:** Minna Mutka, Kristiina Joensuu, Marja Heiskala, Mine Eray, Päivi Heikkilä

**Affiliations:** 1 Department of Pathology, HUSLAB, Helsinki University Hospital and University of Helsinki, FIN-00290 Helsinki, Finland; 2 University of Helsinki, FIN-00290 Helsinki, Finland

**Keywords:** CCR5, Siglec-15, PD-L1, Breast carcinoma, Core needle biopsy

## Abstract

Core needle biopsies (CNB) are widely used to diagnose breast cancer, but the procedure is invasive and thus, it changes the tumor microenvironment. The purpose of this study is to see how the expression of three potentially anti-inflammatory molecules, namely, programmed death-ligand 1 (PD-L1), sialic acid-binding immunoglobulin-like lectin-15 (Siglec-15), and C-C chemokine receptor-5 (CCR-5), are expressed in CNB and surgical resection specimens (SRS). To do this, we compared the amounts of tumor-infiltrating lymphocytes and the levels of CCR5, Siglec-15, and PD-L1 in tumor cells and inflammatory cells as assessed by immunohistochemistry in CNB and the corresponding SRS of 22 invasive breast carcinomas of no special type and 22 invasive lobular carcinomas. The Siglec-15 H-score was higher in tumor cells in the SRS than in the CNB groups. There was no change in tumor cells CCR5 or PD-L1 between CNB and SRS. The positive inflammatory cell numbers for all markers rose between CNB and SRS, as did the amount of Tils. Furthermore, higher grade tumors and tumors with a high proliferation rate had more inflammatory cells that were positive for the markers and also more PD-L1+ tumor cells. Although changes in inflammatory cells can partly be attributed to the larger sample size of operation specimens, the differences also mirror a true change in the tumor microenvironment. The changes in inflammatory cells could be partly due to the need to restrict excess inflammation at the site of the biopsy.

## Introduction

Core needle biopsies (CNB) are the recommended means of diagnostics in breast cancer [1] as they have excellent sensitivity and specificity. They also have the potential to be used in assessing prognostic and predictive factors preoperatively [2]. However, CNB is an invasive procedure that inevitably alters the site from which they are taken. The most known problem is the seeding of tumor cells into the biopsy canal, which could potentially lead to recurrences. However, studies have shown that CNB procedures are not associated with an elevated risk of recurrence or a worse prognosis [3–5]. Nevertheless, changes in the tumor stroma do encompass hemorrhage, fat necrosis, fibrosis, and recruitment of inflammatory cells [6, 7].

The amounts of tumor-infiltrating lymphocytes (Tils) tend to be lower in CNBs than in the corresponding surgical resection specimens (SRS) [8, 9].

In a study using a mouse model, there were fewer CD4+, CD8+, and NK-cells and more myeloid-derived suppressor cells in the tumor stroma in SRS than for CNB. The epithelial-mesenchymal transition pathway and many cytokines were upregulated. All these changes created a prometastatic and immunosuppressive microenvironment in the tumor [10]. Another study showed that CNB also promoted epithelial-mesenchymal transition [11]. Such alterations of the tumor microenvironment could have adverse implications for the patient and thus are an important topic to study.

Programmed death-ligand 1 (PD-L1) is a cell membrane protein that interacts with programmed death-1 (PD-1). It is found in inflammatory cells such as lymphocytes and macrophages. The function of the PD-1/PD-L1 pathway is to limit excess inflammation and prohibit reactions to self-antigens by inducing immune evasion. However, tumor cells can also activate the pathway, and this serves as a way to escape anti-tumor immune responses [12, 13].

Sialic acid-binding immunoglobulin-like lectin-15 (Siglec-15) is another known pathway that evades antitumor immune responses. It is a transmembrane protein on macrophages, dendritic cells, and osteoclasts [14], which binds to target cell sialic acids, thus recognizing them as self and thereby suppresses the T cells [15]. However, tumor cells tend to have a lot of sialic acids on their surface, and their binding to Siglec-15 is a potential mechanism of tumor immune escape [15–17]. Moreover, Siglec-15 seems to be independent of PD-L1-mediated immune escape, and thus is a promising,

potential therapeutic target for PD-L1 treatment-resistant cancers [15, 18].

C-C chemokine receptor-5 (CCR5) is a receptor that is normally found on T-cells, macrophages, dendritic cells, eosinophils, and microglia. CCR5 functions in the recruitment of inflammatory cells at the site of inflammation [19]. It is often overexpressed in breast cancer cells, especially in HER2+ and triple-negative breast cancer and is associated with greater invasiveness and metastatic potential [20, 21]. It also augments tumor growth, facilitates building an immunosuppressive tumor microenvironment, enhances angiogenesis, and induces drug resistance [19, 22–26].

A common feature of these three markers is they are either already used as therapeutic targets in breast cancer [27] or are promising new targets [15, 26]. In particular, the targeting of Siclec-15 might be an alternative pathway to PD-L1 and thus could be an alternative to PD-L1 targeted-therapy–resistant tumors [15], whereas CCR5 targeting seems to be synergistic with PD-L1 targeting [28].

In this study, we have compared the levels of Tils, CCR5, Siglec-15, and PD-L1 in CNB and the corresponding SRS of 22 invasive breast carcinomas of no special type (IC-NST) and 22 invasive lobular carcinomas (ILC). The purpose of this study was to see if the tumor microenvironment changes after CNB. Knowledge of how the procedure itself changes the microenvironment of a tumor should provide valuable information on baseline changes for window of opportunity studies. In such studies, a medical intervention is made between CNB and the operation to see if there are any changes induced by the medications [29].

## Methods

### Patients and tissue samples

The material comprised 44 cases of primary breast cancer, of which 22 were IC-NSTs and 22 ILCs. For detailed information about the cases, see Table 1. The CNB formalin-fixed, paraffin-embedded (FFPE) whole tissue sections and the corresponding SRS FFPE whole tissue sections were collected from the archives of the Department of Pathology at the University Hospital of Helsinki, and all specimens were taken in 2016. The numbers of CNB cores taken per case varied from 2 to 9; the mean was 4.34 cores per patient. The mean time between CNB and operation was 27.5 days and the range 7–82 days. None of the patients received any neoadjuvant therapy.

**Table 1:** Clinicopathologic information about the cases

Factor	IC-NST *n* = 22	ILC *n* = 22
ER status		
Positive	19	22
Negative	3	0
PR status		
PR positive	17	20
PR negative	5	2
Ki67 status		
Low	8	17
High	14	5
HER2 status		
Positive	4	22
Negative	18	0
Tumor type		
ER+HER2−	16	22
ER+HER2+	3	0
ER−HER2+	1	0
TNBC	2	0
Grade		
G1	5	4
G2	6	14
G3	11	4
Lymph node status		
Positive	12	13
Negative	9	9
Size of tumor		
≤ 20 mm	12	11
> 20 mm	10	11
Age of patient		
≤ 50	5	3
51–70	5	8
> 70	12	9

*IC-NST* invasive breast carcinoma of no special type, *ILC* invasive lobular carcinoma, *ER* estrogen receptor, *PR* progesterone receptor, *HER2* human epidermal growth factor receptor 2, *TNBC* triple-negative breast cancer, *G* grade

### Immunohistochemistry

Formalin-fixed paraffin-embedded tissue blocks were cut into 4-μm-thick sections.

After deparaffinization, the slides for Siglec-15 and CCR5 were pretreated in a PT module (LabVision UK Ltd., Suffolk, UK) in Tris-EDTA pH 9.0 (100 °C for 24 min) and cooled to room temperature. Immunohistochemical staining was done using the following antibodies: CCR5 (dilution 1:400, clone T21/8) and Siglec-15 (dilution 1:600, polyclonal). The polymer detection kit EnVision (K5007, Dako) was used in a LabVision Autostainer (Thermo Scientific, Fremont, CA).

PD-L1 was stained in Dako Autostainer (Dako/Agilent, Santa Clara, CA) using the SP142 clone (dilution 1:200). Pretreatment was performed in a PT module in Tris-EDTA at low pH (97 °C for 20 min). The primary antibody was incubated at 24 °C for 30 min. UltraVision Quanto Detection System HRP (Epredia™ TL-060-QHD) was used for detection.

ER, PR, Ki-67, and HER2 were stained in Ventana Benchmark Ultra (Ventana/Roche, Tucson, AZ) using the following antibodies: ER (RTU, clone SP1), PR (dilution 1:50, clone 16), Ki-67 (dilution 1:100, clone MIB-1), and HER2 (RTU, clone 4B5). Pretreatment was performed with Ventana Cell Conditioning Solution CC1 (Roche, Tucson, AZ) at 98 °C for 64 min. The primary antibodies were incubated as follows: ER at 37 °C/16 min, PR 37 °C/32 min, Her-2 at 36 °C/48 min, and Ki-67 36 °C/32 min. OptiView DAB IHC Detection Kit (760-700 Ventana/Roche) was used for ER, PR, and Her-2 and UltraView DAB IHC Detection Kit (760-500 Ventana/Roche) for Ki-67. The slides were counterstained using Mayer’s hematoxylin and then mounted in a mounting medium.

If positivity was seen in HER2 immunostaining, Inform HER2 Dual ISH in situ hybridization with Ventana Benchmark Ultra (Ventana/Roche, Tuscon, AZ) was used for HER2 gene amplification testing. Triple pretreatment with solutions CC1 at 98 °C for 16 min (950-224, Ventana/Roche), CC2 at 98 °C for 24 min (950-223, Ventana/Roche), and protease-3 at 37 °C for 16 min (780-4149, Ventana/Roche) was done. The HER2 gene was targeted using a dinitrophenyl-labeled probe and the chromosome 17 centromere was targeted with a digoxigenin-labeled probe (INFORM HER2 Dual ISH DNA Probe Cocktail, 780-4422, Roche/Ventana/Tuscon, AZ, USA 780–4422). HER2 was visualized as black signals with VENTANA ultraView Silver ISH DNP (SISH) Detection (760-098, Roche/Ventana/Tuscon, AZ, USA) and Chr17 as red signals with VENTANA ultraView Red ISH DIG Detection (780-4422, Roche Ventana Tuscon, AZ, USA).

**Fig. 1:**
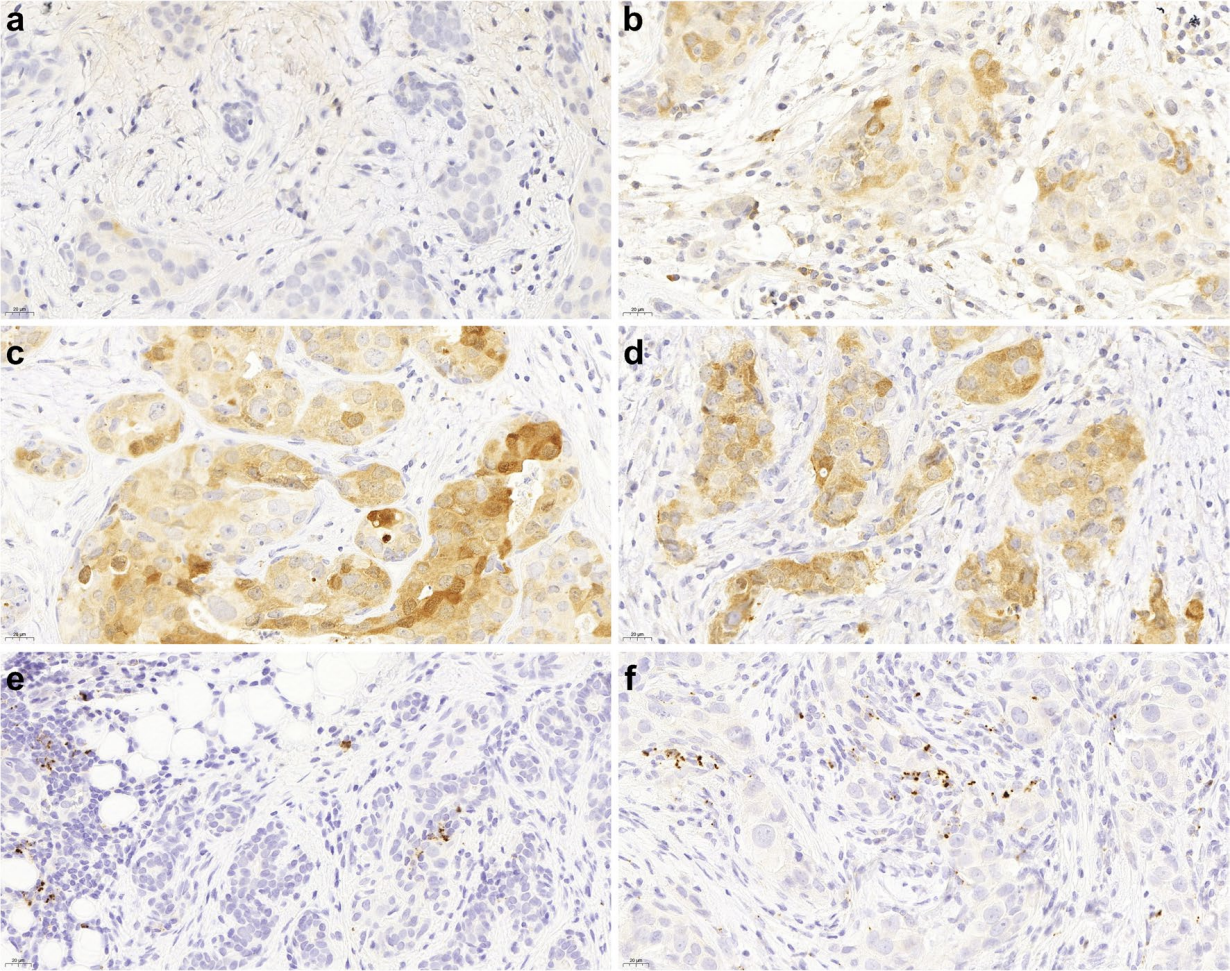
All immunohistochemical stainings of a CNB-SRS pair. **a** CCR5 of the CNB. There were virtually no positive inflammatory cells in the CNB, and the tumor cell H-score was 53. Tumor cell positivity is cytoplasmic. **b** In the SRS, there were positive inflammatory cells (score 2%) and the H-score had risen to 130. Inflammatory cell positivity is cytoplasmic. **c** The tumor cell Siglec-15 H-score was 165, but there were no positive inflammatory cells. Tumor cell positivity is cytoplasmic. **d** The H-score rose to 180 in the SRS, and there were some positive immune cells (score 2%). Inflammatory cell positivity is cytoplasmic. **e** PD-L1 of the CNB. There was no tumor positivity, but the inflammatory cell score was 2%. Inflammatory cell positivity is cytoplasmic. **f** In the SRS, the inflammatory cell score was 10%, and the tumor cells remained negative

Tumor cell positivity was determined for Siglec-15, CCR5, and PD-L1. Tumor cell positivity for Siglec-15 and CCR5 was determined as follows: an H-score was given by adding 1 × the percentage of weakly positive tumor cells, 2 × the percentage of moderately positive tumor cells, and 3 × the percentage of highly positive tumor cells [30]. In contrast, to measure PD-L1, a percentage of positive tumor cells were used. A score was also given for inflammatory cell positivity for all three markers. The score was defined as the percentage of tumor stromal area covered by positive immune cells. Figure 1 shows all the immunohistochemical stainings in both the CNB and the SRS of one case.

All CNBs and SRSs were also assigned a Tils percentage in H&E-stained sections according to the guidelines of the International Immuno-Oncology Biomarkers Working Group, https://www.tilsinbreastcancer.org/ [31, 32]. In short, the percentage of stromal area inside invasive tumor borders occupied by mononuclear inflammatory cells was obtained.

### Statistics

Statistical analyses were performed using SPSS 22.0 for Windows (SPSS Incorporation, Chicago, IL, USA). The continuous changes in the markers comparisons for CNB and SRS were tested using the paired-samples *t* test, whereas comparisons of the IC-NSTs and ILCs were made by the Wilcoxon matched-pair signed-rank test. The Kruskal-Wallis test and the Mann-Whitney *U* test were used for comparing categorical differences between histological types and differences in the changes in the markers between histological type, grade, and Ki-67 status. Ki67 high was defined as > 20%, and low as ≤ 20%. The percentages of Tils were considered low for 1–10%, moderate for 11–49% and high for 50–100%. Probability values *p* < 0.05 were considered to be significant.

## Results

### Description of staining patterns

The Tils of the H&E sections were evenly distributed in 29 cases and patchy in 15 cases. Tertiary lymphoid structures were seen in 9 cases. A biopsy canal was clearly visible in 17 out of 44 SRSs. In the rest of the cases, the biopsy canal could not be seen with certainty in the sections used. A comparison was done between Tils percentages within the biopsy canal and distal to the canal. The mean Tils value within the canal was 10.41 and distal to the canal 14.53. For the rest of the results, a Tils count excluding the biopsy canal was used in the cases where the canal was visible.

CCR5 staining was cytoplasmic in both tumor cells and inflammatory cells. Tumor cell positivity was generally evenly distributed, but the intensity of the positivity varied in different areas; thus, it was more intense in the periphery of the tumor in 29 cases and in the center in one tumor, whereas in 12 cases, there was no difference between the center and the periphery. As the amounts of positive inflammatory cells were generally low, the staining was mostly patchy. The mean H-score of CCR5 was 99.59 within the biopsy canal and 96.29 distal to it, and the mean percentage of positive inflammatory cells was 1.50 within the canal and 1.31 distal to it.

Siglec-15 staining was cytoplasmic in both tumor cells and inflammatory cells. Tumor cell positivity was mostly evenly distributed. However, in five cases, the periphery stained more intensely than more central regions of the tumors. The staining was patchy as the amounts of positive inflammatory cells were generally low. The mean H-score of Siglec-15 was 72.65 within the biopsy canal and 71.88 distal to it.

PD-L1 staining was cytoplasmic in both tumor cells and inflammatory cells. The positivity for both cell types was patchy. Half of the cases showed only a few positive patches and half several positive patches for both tumor cells and inflammatory cells. The mean percentage of positive tumor cells within the biopsy canal was 0.35 and inflammatory cells 2.59; distal to the canal, the percentages were 0.94 for tumor cells and 5.47 for inflammatory cells.

### Tumor Siglec-15 H-scores are higher in the SRS group

Changes in the marker scores in tumor cells between CNBs and SRSs are shown in Table 2.

**Table 2:** Changes in tumor cell positivity between core needle biopsy (CNB) and surgical resection specimen (SRS) and the *p*-values for the significance of the change

Marker	IC-NST	*p*-value	ILC	*p*-value	All carcinomas	*p*-value
CCR5 H-score for CNB	91.32	0.935	58.82	0.330	75.07	0.473
CCR5 H-score for SRS	89.91		77.91		83.01	
Mean change CCR5	− 1.41		19.09		8.84	
Siglec-15 H-score for CNB	65.91	0.030*	42.09	0.092	54.00	0.012*
Siglec-15 H-score for SRS	84.32		70.45		77.39	
Mean change Siglec-15	18.41		28.36		23.39	
PD-L1+ tumor cells % for CNB	2.05	0.344	0.35	0.574	1.20	0.973
PD-L1+ tumor cells % for SRS	2.24		0.19		1.21	
Mean change PD-L1	0.19		− 0.16		0.01	

*IC-NST* invasive breast carcinoma of no special type, *ILC* invasive lobular carcinoma, *significant changes

There was a significant rise in the Siglec-15 H-score in the SRS compared to the CNB groups. When individually assessed, this rise was significant for the IC-NSTs, but only approached significance for the ILCs. The differences in the changes of the scores were not significant between IC-NSTs and ILCs. There were no significant differences in the H-scores between IC-NSTs and ILCs.

There were no significant changes between CNB and SRS groups for either the CCR5 tumor H-scores or the PD-L1+ tumor cells percentages, nor were there any differences in the changes between IC-NSTs and ILCs. The CCR5 H-scores were not associated with differences in tumor histology. There were significantly more PD-L1+ tumor cells in the SRS of the IC-NSTs compared to those of the ILCs, but this difference was not seen for the CNB group.

### There are more CCR5+, Siglec-15+, and PD-L1+ inflammatory cells in SRS

The changes of the marker scores in inflammatory cells between CNB and SRS groups are shown in Table 3.

**Table 3:** Changes in inflammatory cell positivity between core needle biopsy (CNB) and surgical resection specimen (SRS) and the *p*-values for the significance of the change

	IC-NST	*p*-value	ILC	*p*-value	All carcinomas	*p*-value
Tils % for CNB	18.91	0.036*	4.09	0.025*	11.50	< 0.001*
Tils % for SRS	26.27		6.36		16.32	
Mean change Tils	7.36		2.27		4.82	
CCR5+ inflammatory cells % for CNB	0.55	0.001*	0.23	0.002*	0.39	< 0.001*
CCR5+ inflammatory cells % for SRS	2.18		1.36		1.77	
Mean change CCR5	1.64		1.14		1.39	
Siglec-15 inflammatory cells % for CNB	0.55	0.001*	0.18	0.012*	0.37	< 0.001*
Siglec-15 inflammatory cells % for SRS	2.50		0.81		1.67	
Mean change Siglec-15	1.95		0.62		1.30	
PD-L1+ inflammatory cells % for CNB	1.94	< 0.001*	0.70	0.001*	1.32	< 0.001*
PD-L1+ inflammatory cells % for SRS	8.32		2.12		5.22	
Mean change PD-L1	6.38		1.41		3.90	

*IC-NST* invasive breast carcinoma of no special type, *ILC* invasive lobular carcinoma, *significant changes

There was a significant rise in the percentage of Tils between the CNB group and the SRS group. This was also seen for both IC-NSTs and ILCs, but the difference in the change for IC-NSTs and ILCs was not statistically significant (*p* = 0.548). There were significantly higher percentages of Tils in IC-NSTs than in ILCs in both the CNB group (*p* = 0.003) and the SRS group (*p* = 0.009).

There were significant rises in the scores for CCR5+, Siglec-15+, and PD-L1+-inflammatory cells of the SRS group, and these increases were seen for both the IC-NSTs and ILCs. However, the changes were larger for the IC-NSTs for both Siglec-15 (*p* = 0.012) and PD-L1 (*p* = 0.002). The CCR5 scores were similar for IC-NSTs and ILCs.

There were significantly more PD-L1+ inflammatory cells in the IC-NSTs than in the ILCs for both the CNB (*p* = 0.002) and SRS (*p* = 0.002) groups. A similar difference was also seen for Siglec-15 in the SRS group (*p* = 0.001).

### PD-L1 inflammatory cell positivity rises with Tils percentage

In both the CNB and the SRS groups, there was more positivity for PD-L1 in inflammatory cells in the tumors that had higher amounts of Tils (Table 4). A similar relationship was seen between CCR5-positive inflammatory cells and Tils.

**Table 4:** Influence of Ki-67 and grade (G) to inflammatory cells

	Tils CNB	*p*-value	Tils SRS	*p*-value	CCR5 CNB	*p*-value	CCR5 SRS	*p*-value	Siglec-15 CNB	*p*-value	Siglec-15 SRS	*p*-value	PD-L1 CNB	*p*-value	PD-L1 SRS	*p*-value
Ki-67																
Low	4.40	0.002*	6.20	0.002*	0.16	0.10*	1.20	0.006*	0.08	0.002*	1.12	0.012*	0.74	0.019*	2.08	0.001*
High	20.84		29.63		0.68		2.53		0.74		2.44		2.08		9.34	
Grade																
G1	3.56	0.005*	3,67	0.008*	0.00	0.020*	1.00	0.010*	0.11	0.005*	1.33	0.037*	0.44	0.060	1.72	0.020*
G2	7.70	G1–G3 0.016*	11.65	G1–G3 0.007*	0.25	G1–G3 0.029*	1.35	G1–G3 0.023*	0.10	G1–G3 0.047*	1.05	G2–G3 0.035*	0.96		4.10	G1–G3 0.020
G3	21.33	G2–G3 0.019*	30.13		0.80		2.80	G2–G3 0.037*	0.87	G2–G3 0.007*	2.79		2.33		8.80	G2–G3 0.050*
Tils																
Low					0.24	0.008*	1.30	0.012*	0.15	0.013*	1.48	0.079	0.46	< 0.001*	2.05	< 0.001*
Moderate					1.17	Low-moderate 0.008*	2.00	Low-high 0.012*	1.00		1.13		4.83	Low-high 0.001*	7.88	Low-moderate 0.007*
High					0.40		3.83		1.00		3.33		2.80	Moderate-high 0.003*	17.50	Low-high < 0.001*

*p*-values for the significance of the differences between Ki-67 high and low tumors, and different grades and different levels of Tils are shown as well as significant *p*-values for pairwise comparisons for grade and Tils. *CNB* core needle biopsy, *SRS* surgical resection specimen, *Tils* tumor-infiltrating lymphocytes, *significant changes

Tumor cell positivity for PD-L1 in the SRS group was higher for the tumors that had higher amounts of Tils (*p* = 0.001). This was seen in pairwise comparisons for both tumors with low amounts of Tils compared to high amounts (*p* < 0.001) and also for moderate amounts of Tils compared to high amounts (*p* = 0.009). A similar relationship was seen in the CNB group (*p* = 0.012) although this relationship was not significant in the pairwise comparisons. No correlation for CCR5 and Siglec-15 in tumor cells with Tils in the SRS group was seen. In the CNB group, however, there was a significantly higher H-score in CCR5 for tumors with higher amounts of Tils (*p* = 0.034), and no significant differences were seen in pairwise comparisons.

The more the Tils percentage rose between CNB and SRS, the more positivity was also seen in PD-L1-positive inflammatory cells (*p* = 0.001). This relationship was not seen for any of the other markers in either the tumor cells or the inflammatory cells.

### Higher grade and Ki-67 high tumors have higher scores of positive inflammatory cells

Table 4 shows the mean positive inflammatory cell percentages for all markers for Ki-67 and for all grades of tumors. Percentages were all higher in the higher-grade tumors and in tumors with a high Ki-67 status. Furthermore, there was a significantly greater rise in PD-L1+ inflammatory cells between the CNB and SRS group for the higher-grade tumors (*p* = 0.020) and in tumors with high Ki-67 status (*p* ≤ 0.001). The pairwise comparisons for grade showed the change was significant between G2 and G3 tumors (*p* = 0.046).

Tumors with higher Ki-67 scores (*p* = 0.017 for CNBs and *p* = 0.001 for SRS) and higher-grade tumors (*p* = 0.003 for CNB and *p* = 0.042 for SRS) also had higher percentages of PD-L1+ tumor cells. The pairwise comparisons showed the differences were significant in the SRS (*p* = 0.036) and CNB (*p* = 0.020) between G2 and G3 and also in the CNB between G1 and G3 (*p* = 0.005). The changes between CNB and SRS were not significantly different.

Tumor cell H-scores for the different markers were not influenced by Ki-67 status or grade to the same extent. The only difference in H-scores was seen for the CCR5 marker in the SRS group (*p* = 0.011), whereby the higher-grade tumors had higher scores. More specifically, this difference was seen only between G1 and G3 tumors (*p* = 0.009).

## Discussion

This study compared Tils and three markers, CCR5, Siglec-15, and PD-L1 in tumor cells, and inflammatory cells in the CNB and SRS of 22 IC-NSTs and 22 ILCs. The purpose was to see how the procedure of CNB changes the tumor microenvironment. A significant rise was seen in Tils and all markers in the inflammatory cells for SRS compared to CNB. Tumor cell PD-L1 or CCR5 did not change significantly, although there was more Siglec-15 positivity in tumor cells in the SRS group.

A previous study has shown that there are higher numbers of Tils in SRS than CNB. The researchers suggested that this could be due to immunoactivation by the CNB procedure, although tumor heterogeneity could not be excluded as an explanation. A rise of Tils was reported to be higher in younger patients and also when there was a long interval between CNB and the operation [8]. Another study showed that the expression of 14 genes changed from CNB to SRS; consequently, these changes indicated that there was a rise in tumor-associated macrophages and immunoactivation in SRS [29].

The upregulation of PD-L1+ inflammatory cells could be due to a need to restrict inflammation at the site of the biopsy. In tumor cells, PD-L1 is intrinsically upregulated whereas its upregulation in inflammatory cells is reactive [12]. The consequences of upregulated PD-L1 for making a prognosis is therefore dual, and the evidence is partly conflicting [12, 13, 33, 34]. For example, some studies report a worse prognosis for tumors with PD-L1 positivity [12, 33]. However, when combined with a high Tils score, PD-L1 is, rather, a marker that indicates an active immune reaction and also is associated with a better prognosis [12, 34]. Therefore, the increases of PD-L1 in the SRS group could be a marker of immunoactivation caused by external stimuli. In this study, the rise in Tils and PD-L1 was similar, which indicates a possible immunoactivation effect.

The changes in Siglec-15+ immune cells could have arisen from the requirement to reduce excess inflammation as this is considered the normal function of the molecule [16]. If this is the case, then the elevated Siglec-15+ immune cell numbers in SRS after CNB could also be considered to be a reaction of the immune system. Unlike for PD-L1, siglec-15+ immune cells did not rise with Tils. The question about any effects of external stimuli on PD-L1 and Siglec-15 and immunoactivation is interesting and requires more research.

The immune cell score of PD-L1 is used as the marker for therapy [27]. The scores tended to be higher in the SRS group in this study; thus, it would seem appropriate to prefer SRS for PD-L1 assessment in cases where there exists an option between CNB and SRS so as not to miss cases for which therapy is indicated. In the setting of neoadjuvant therapy, a CNB is often the only eligible sample, and in these cases, CNB has to be used.

Although Siglec-15 positivity has been reported in tumor cells [14, 35, 36], the significance of this positivity is not clear. The effect of elevated Siglec-15 in tumor cells may vary depending on the immune status of the tumor, but in some instances, it has been found to be a good prognostic sign [37, 38]. However, more research is needed on this topic.

The CCR5+ inflammatory cell count also rose. CCR5 is associated with breast cancer progression [22, 25, 39, 40]. It is overexpressed in many breast cancers, especially basal breast cancer and TNBC [19]. Expression of CCR5 in breast cancer cells in basal breast cancer has been reported to be coupled with increased invasiveness and metastatic potential. In luminal breast cancer cells, CCR5 is associated with altered proliferation [20]. This study reported that breast cancer cell CCR5 H-scores remained at the same level for CNB and SRS.

The whole slides from SRS generally are larger and more representative of the lesion than CNB, which might partly explain the elevated numbers for CCR5+, Siglec-15+, and PD-L1+ inflammatory cells found in the SRS. For example, PD-L1 staining is known to be heterogenous and often underestimated in tissue microarrays (TMAs) [13, 33]. However, there are several studies that compared the concordance of many characteristics of breast carcinoma in CNB and SRS. These studies show that there is generally a good concordance in the evaluation of cancer histology [2], ER status [2, 41–44], and HER2 status [2, 42]. The concordance rate is slightly lower for tumor grade [2, 45] and PR status [2, 42–44]. Even a micro-focal phenomenon such as lymphovascular invasion has a concordance rate of 69% between CNB and SRS [2], and thus, CNB can be considered a good representation of the whole tumor. A study on lymphocyte rate concordance in TMA cores and whole-tumor slides showed a moderate concordance with only one TMA core, but concordance continued to rise for up to four cores [46].

We consider that the sampling methodology difference between CNB and SRS is not sufficient to explain all the changes seen in this study. However, for inflammatory cells and PD-L1+ tumor cells, where staining was generally patchy, heterogeneity can at least partly explain the differences. For Siglec-15 and CCR5 tumor cells the staining pattern was more even, although the intensity of the staining varied and tended to be higher in the periphery. In previous studies, sampling accuracy had been improved when four or more biopsy cores were obtained [42]. In this study, the mean core number was 4.34, and generally, the CNB provided ample tumor material which should lessen the effect of tumor heterogeneity.

Previous studies on the hormone receptor staining showed generally higher staining positivity in CNB than operation specimens, which was probably due to better fixation [2, 41]. In our present study, staining was higher in the SRS group. However, as mentioned above, the staining is expected to work better in CNB compared to operation specimens, which implies that the changes observed in our study cannot only be attributed to sample size per se.

We also compared the markers with the tumor Ki-67 status and grade and found that there were more positive inflammatory cells in higher grade tumors and the Ki-67-high tumors, and that there were also more PD-L1+ tumor cells. These elevated scores were seen in both the SRS and the CNB group. This is a well-known phenomenon and has been reported in previous studies [12, 33, 47, 48].

This study has all the known limitations of a retrospective study. Another limitation is the use of the SP142 antibody for PD-L1 detection, as this antibody is known to give lower scores than other corresponding antibodies [49]. However, SP142 was chosen for this study as it still is the only antibody approved for diagnostic use in metastatic breast cancer in Finland. Caution must be used in the interpretation of PD-L1 results as some positivity might have been missed.

The main conclusion of this study is that Tils, CCR5, Siglec-15, and PD-L1 are all higher at SRSs than their corresponding numbers at CNBs. Although this difference can partly be assigned to the greater sample size of SRSs and by tumor heterogeneity, it could also mirror a true immunoactivation caused by the procedure of CNB. This putative immunoactivation effect is an interesting phenomenon and warrants more research.

## Data Availability

The datasets generated during and/or analyzed during the current study are available from the corresponding author on reasonable request.
